# Development of a questionnaire to assess maternal attitudes towards infant growth and milk feeding practices

**DOI:** 10.1186/1479-5868-8-35

**Published:** 2011-04-21

**Authors:** Rajalakshmi R Lakshman, Jill R Landsbaugh, Annie Schiff, Wendy Hardeman, Ken K Ong, Simon J Griffin

**Affiliations:** 1 MRC Epidemiology Unit, Cambridge, UK; 2 Department of Public Health and Primary Care, University of Cambridge, UK; 3 Department of Paediatrics, University of Cambridge, UK; 4 UKCRC Centre for Diet and Activity Research, Cambridge, UK

**Keywords:** infant feeding, nutrition, growth, attitudes, obesity prevention

## Abstract

**Background:**

There is increasing recognition that public health strategies to prevent childhood obesity need to start early in life. Any behavioural interventions need to target maternal attitudes and infant feeding practices, This paper describes the development and preliminary validation of a questionnaire to assess maternal attitudes towards infant growth and milk feeding practices.

**Methods:**

We designed a 57-item (19 questions), self-administered questionnaire to measure the following four domains- 1) type of milk feeding, decision making and sources of advice; 2) frequency and quantity of milk feeds; 3) attitudes to infant feeding and growth; and 4) theory-based beliefs about following infant feeding recommendations. Forty mothers completed the questionnaire on two occasions six days apart (to assess test-retest reliability) and then participated in a semi-structured, open-ended telephone interview covering the same domains (to assess criterion validity). Percentage agreement, Cohen's Kappas (for categorical variables) and Spearman's correlation coefficients (for continuous variables) were used to quantify reliability and validity. Internal consistency between theory-based constructs (self-efficacy, outcome expectancy and intention) was quantified by Chronbach's alpha.

**Results:**

Of the 57 questionnaire items 51 (89%) had percentage agreement above 70% indicating good test-retest reliability, and the remaining 6 items had moderate or substantial levels of agreement (kappa 0.41-0.68). Comparing questionnaire with interview coding (validity), percentage agreement was above 66% for 39/57 items (68%). Of the 16 items with percentage agreement below 66%, only five had kappa values below 0.20 (two items had insufficient interview responses). Internal consistency was 0.51, 0.79 and 0.90 for self-efficacy, outcome expectancy and intention respectively.

**Conclusions:**

This questionnaire could be a useful tool in understanding the determinants of infant feeding and the 'causal mechanism' of interventions that target infant feeding practices to prevent early obesity.

## Background

Rapid weight gain during infancy is recognised as an important risk factor for later obesity[1-3]. Most parents are poor at recognising overweight and obesity in their children[4-7] and one in five children in England are already overweight when they start school[8]. Hence early prevention of obesity has become a national priority[9,10]. Informed by the MRC framework for complex interventions[11], we are developing an intervention to reduce formula-milk intake and prevent excess weight gain during infancy which we plan to test in a randomised controlled trial. Systematic reviews of the literature demonstrated gaps in the evidence-base for interventions in this area[12,13] and particularly in our understanding of how parents make decisions concerning the frequency and quantity of infant milk feeds[14]. Although other questionnaires have been developed to assess child[15,16] and infant[17] feeding practices, there are no instruments to assess milk feeding practices in younger children, hence we developed a questionnaire to identify correlates of infant milk feeding practices. The questionnaire could also be used to assess the effectiveness and mechanism of action of interventions to optimise growth and nutrition during infancy. The objectives of this paper are to describe the process of developing the questionnaire and its preliminary testing. We also describe maternal attitudes and milk feeding practices in this small sample.

## Methods

### Questionnaire development

The purpose of the questionnaire was to assess maternal infant milk feeding practices, mothers' decision making regarding how much and how often to feed their babies, their attitudes to infant feeding and growth, and beliefs about following infant feeding recommendations. The 57-item (19 questions), self-administered questionnaire was developed after conducting literature reviews[18-21] and a qualitative study using a flexible semi-structured interview schedule involving 38 parents (n = 35 mothers). Three main themes emerged on parents' decision-making regarding volume and frequency of formula-milk feeds; i) baby's appetite (if the baby finished the bottle, or cried between feeds, more was added to the next feed), ii) instructions on formula milk tins/packets (if the baby did not take what it said on the tin, they were offered a feed again after a short gap) iii) baby's growth (as baby's weight increased, feeds were increased). Parents got information on bottle-feeding from various sources -family, friends, other mothers, 'parent support groups', books, magazines, the internet, formula-milk packets, supermarket shelves, health visitors and midwives. The main barriers to reducing formula-milk feeds were concerns that the baby would cry, be hungry, wake up at night and demand more frequent feeds.

An iterative process was used and numerous revisions were made in response to input from groups of researchers and mothers. Content validity was assessed by extensive pilot testing with mothers (n = 60) participating in a birth cohort study (The Cambridge Baby Growth Study)[22]. Questions covered four domains: 1) type of milk feeding, decision making, and sources of advice, 2) frequency and quantity of feeds, 3) attitudes to infant feeding and growth, and 4) theory-based beliefs about following recommendations to reduce formula-milk feed quantities.

#### Type of milk feeding, decision making, and sources of advice

Questions related to the type of milk feeding: breastmilk, expressed breastmilk, formula-milk feeds, and type and brand of formula-milk feeds. Regarding decision making about frequency of milk feeds, the question was phrased '*When deciding how often to feed your baby, do you usually... feed your baby on demand, or follow a routine, or do a combination of both?' *Regarding decision making on quantity of milk feeds, our qualitative study showed that mothers either followed the guidelines printed on the formula-milk packaging, or based their decisions on the baby's appetite or growth. Hence our question was phrased '*When deciding how much to feed your baby, do you usually...follow guidance, or depend on baby's appetite, or depend on baby's growth?'*

Our systematic review[14] reported that mistakes in preparation of formula-milk feeds with formula-milk powder were common. Parents sometimes heaped or tightly packed the scoops or added powder to the bottle first which resulted in an over-concentrated feed. Furthermore parents reported that they did not receive sufficient advice from healthcare providers. Consequently, we included questions on how feeds were prepared, mothers' sources of advice, and which advice was followed. These questions would also be of particular value to the process evaluation of any trial to change parents' infant feeding behaviour.

#### Frequency and quantity of feeds

Amount of milk intake may change often during infancy and therefore to quantify the association between milk intake and infant growth, it may be necessary to assess milk intake frequently (every 4-6 weeks in the first six months). A 4 day diet diary would be burdensome for mothers of newborn infants to complete frequently, hence the following questions were developed as a pragmatic substitute. Example questions include '*In a typical 24 hour period how much formula milk does your baby have? Amount of formula milk per feed? Number of formula feeds per day? Number of scoops of formula milk powder per feed? Duration of typical daytime feed?' *Similar questions were included for breastfeeds, water and other drinks. In order to assess whether milk feeds were replaced by solid/semi-solid foods, we included questions relating to these, for example '*What was your baby's age in months when you started solid/semi-solid food?'*

#### Attitudes to infant feeding and growth

A literature review identified validated questionnaires on breastfeeding self-efficacy[18-21] and self-efficacy in infant care[23,24]. These were used to develop questions to assess mother's confidence (self-efficacy)[25] in infant growth monitoring and feeding so that her baby would not gain too much weight. The questions included eight items, each scored on a five-point Likert-type scale from 'strongly agree' to 'strongly disagree'. For example '*I am confident that I can feed my baby so they do not gain too much weight'*. A single question on perception of size -'*Do you think your baby is... underweight, OR about right, OR overweight?'*, was derived from a published study[26].

#### Theory-based beliefs about following recommendations to reduce formula-feed quantities

In 2004 the World Health Organisation (WHO) and other international bodies reduced their recommendations for energy requirements during infancy from previous (1985) recommendations[27]. Mothers' beliefs about following these new recommendations would influence whether the recommendations were followed. The 11 items in this domain were chosen to measure self-efficacy (confidence in performing a behaviour and overcoming barriers to that behaviour), outcome expectancies (expectation that a positive outcome will occur as a function of that behaviour)-the hypothesised mediators of behaviour change according to Social Cognitive Theory[28,29], and intentions as informed by Theory of Planned Behaviour[30]. These could be scored on a five-point Likert-type scale from 'strongly agree' to 'strongly disagree'. Through our qualitative study of 38 mothers we identified the most common barriers to reducing formula-milk feed quantities (baby would cry, remain hungry, wake up frequently), and used these to create the items in this scale. We also wanted to measure the three dimensions of outcome-expectancies (physical, social and self-evaluative) which predict behaviour[29]. The items were worded positively and negatively and presented in random order. For example '*If I follow the new feeding recommendation, my baby will wake up frequently at night' *(negative physical outcome expectancy).

### Procedures

Thirty one mothers were recruited from an ongoing birth cohort study[22]. To include a more diverse range of participants, we also recruited nine exclusively formula-milk feeding mothers from a focus group study conducted to inform intervention development. To assess test-retest reliability, following receipt of a completed postal questionnaire, the same questionnaire was posted and mothers were asked to complete it a second time (median time interval between completion of the two questionnaires was 6 days (range 2-16 days).

To validate the questionnaire, no 'gold-standard' existed, therefore semi-structured interviews were used to assess criterion validity as previously reported for the validation of a questionnaire covering correlates of children's physical activity[31]. We developed an open-ended, semi-structured interview schedule in which participants could make general comments on the questionnaire, clarify the meaning of the questions, and expand on their answers, giving a richer response (see Additional file 1 for interview schedule). After the first questionnaire was returned, an appointment was made for a face-to-face or telephone interview with the mothers. At the start of the interview, it was confirmed that mothers had completed the second questionnaire and they were asked for general comments on the questionnaire. The median time interval between completion of the first questionnaire and interview was 8 days (range 3-16 days). Interviews were conducted by RL or JL, were tape-recorded and were transcribed by an independent company. The interview data were used as the criterion measure against which to compare the participant's first questionnaire responses. For each participant, two researchers (RL and JL) used the transcripts to fill in a blank questionnaire. This was done independently and blind to the participant's questionnaire responses. For the two questions (questions 16 and 19 with a total of 19 items) which were scored on a five-point Likert type scale, the transcripts were used to mark a collapsed three-point scale 'agree, neutral, disagree'. Once all of the transcripts were coded, the two researcher-completed questionnaires (for each participant) were compared, and in the event of disagreement, consensus was reached by discussion. This final 'agreed' (by both researchers) questionnaire was used as the 'criterion' for comparison against the participant's first questionnaire responses to assess criterion validity.

Approvals for the study were obtained from the local Research Ethics Committee and research governance committees of the local hospital and Primary Care Trust. Demographic details and written informed consent were obtained from all participants.

### Statistical Analysis

All analyses were performed using Stata version 10.1 (Stata Corp LP, College Station, Texas). The test-retest reliability (between 1^st ^and 2^nd ^questionnaires) and criterion validity (between 1^st ^questionnaire and interview coded response) were assessed for each item by calculating percentage exact agreement and chance-corrected agreement (Cohen's kappa). A percentage agreement ≤ 66% was used to indicate fair agreement [32,33] and kappa values were categorized as: poor (< 0.0), slight (0.00-0.20), fair (0.21-0.40), moderate (0.41-0.60), substantial (0.61-0.80), and almost perfect (0.81-1.0) agreements[34]. Weighted kappas were calculated for ordinal categorical variables and Spearman's correlation coefficients for continuous variables.

To assess internal consistency we calculated Cronbach's alpha for items measuring theory-based constructs- self-efficacy, outcome-expectancies and intentions. Negatively worded items were re-coded. A summary score for each construct was calculated by summing the individual item scores and dividing by the number of items in the construct (4 for self-efficacy, 5 for outcome-expectancies and 2 for intentions). The correlations between a summary score for these constructs were calculated.

## Results

Forty mothers completed the first questionnaire; of these two did not complete the 2^nd ^questionnaire and two others did not complete the interview. The mean age of mothers was 34 years (range 21-42 years) and the average age of their babies was 5.2 months (range 3.3-8.7 months, 55% girls). Eighty percent of the mothers had one child, 7.5% had two children and 10% had three children (1 participant -missing data). The majority were White (95%), 58% were professionals and 70% had degree level or higher qualifications. Ten percent of mothers were exclusively breastfeeding, 55% feeding exclusively formula-milk and 35% combined breastfeeds and formula-milk feeds.

### Overall results

The inter-rater agreement between the two researchers coding the interviews was high for 56/57 items (agreement above 80%, kappa above 0.6; data not shown). Overall, for test-retest reliability, percentage agreement was good (above 66%) for 89% (51/57) of the items. For the remaining 6 items (percentage agreement between 47% and 66%), Cohen's kappa's were moderate or substantial (0.41-0.68). Overall, for validity, percentage agreement was above 66% for 68% (39/57) of the items. Two items had insufficient data to estimate percentage agreement and of the remaining 16 items, only five had kappa values less than fair (< 0.20).

### Type of milk feeding, decision making, and sources of advice (Table 1)

**Table 1:** Type of milk feeding, decisions and advice (21 items)

Question wording		**Test-retest reliability**^**a**^		**Validity**^**b**^		**Frequency**^**c**^
**What feeding methods are you currently using? **(n = 40)	**Question**^d^	**%Agreement**^e^	**Kappa**^f^	**%Agreement**^e^	**Kappa**^f^	**n(%)**
Breast milk directly from the breast	1a	100.0	1.00	100.0	1.00	18(45)
Expressed breast milk from bottle	1b	97.3	0.91	91.9	0.68	7(18)
Formula milk, made up from powder or concentrate	1c	100.0	1.00	100.0	1.00	25(63)
Ready-to use formula milk	1d	91.9	0.79	75.7	0.24	12(30)
Others	1e	86.5	0.47	86.5	0.25	7(18)
**When deciding how often to feed your baby, do you usually? **(n = 40)						
A)Feed your baby on demand	2a	88.9	0.75	75.7	0.49	14(35)
B) Follow a routine	2b	91.4	0.72	83.8	0.42	9(23)
Do a combination of A and B	2c	82.9	0.66	70.3	0.40	21(50)
**When deciding how much to feed your baby, do you usually? **(n = 40)						
Follow guidance	17	92.1	0.36	91.9	-0.04	3(7)
Depend on baby's appetite						37(93)
Depend on baby's growth						0(0)
**Which brand, and type of formula milk do you usually use? **(n = 26)						
Brand	6a	87.5	0.83	87.5	0.83	
Type	6b	82.6	0.71	79.0	0.65	
**If you make up formula from milk powder, are the scoops usually? **(n = 25)						
rounded/heaped OR flat	11a	92.7	0.85	Insufficient responses		0(0)
loosely packed OR tightly packed	11b	78.3	0.56	54.6	0.07	14(56)
do you add powder first OR water first	11c	100.0	1.00	100.0	1.00	1(4)
**If you received/read advice on formula** **feed preparation, we would like to know which advice you followed**. (n = 25)						
Midwife	12a	78.3	0.57	66.7	0.23	9(36)
Health visitor	12b	73.9	0.59	45.8	0.07	12(48)
Friends	12c	87.0	0.76	50.0	0.11	13(52)
Family	12d	78.3	0.59	41.7	0.02	13(52)
Formula milk packet or tin	12e	73.9	0.55	62.5	0.32	22(88)
Leaflet	12f	90.9	0.81	75.0	0.10	6(24)
Other	12 g	100.0	1.00	95.0	0.77	2(10)

^a^Test-retest reliability-1^st ^and 2^nd ^questionnaires

^b^Validity-1^st ^questionnaire and interview

^c^Frequency of respondents 1^st ^questionnaire - for Question 11a- answering scoops rounded/heaped; 11b loosely packed; 11c added powder first. For Question 12a-12 g answering followed all or some of the advice.

^d^Question- Question number

^e^Agreement- Percentage exact agreement- over 66% good agreement

^f^Kappa- Chance corrected agreement or Cohen's Kappa- < 0 poor; 0-0.20 slight; 0.21-0.40 fair; 0.41-0.60 moderate; 0.61-0.8 substantial; 0.81-1 almost perfect

All 21 items in this domain had percentage agreement over 73% for test-retest reliability and 71% of the items had percentage agreement over 66% for validity. Of the six items that had less than 66% agreement for validity, four related to what advice was followed and two related to how feeds were prepared.

Regarding decisions on the frequency of feeds, 50% used a combination of demand and routine feeding. When deciding on the quantity of feeds, 93% were led by the baby's appetite. Sixty-three percent of all mothers (25/40) prepared formula-milk from powder or concentrate and of these 88% (22/25) reported following the advice on the packaging about feed preparation. However, 44% (11/25) of mothers who prepared formula-milk from powder reported that they tightly packed the scoops.

### Frequency and quantity of feeds (Table 2)

**Table 2:** Frequency and quantity of feeds (16 items)

Question wording		**Test-retest reliability**^**a**^			**Validity**^**b**^				
	**Question**^**c**^	**Corr**^**d**^	**%Ag**^**e**^	**Kap**^**f**^	**Corr**^**d**^	**%Ag**^**e**^	**Kap**^**f**^	Median	Range
**Breastfeeding**									
In a typical 24 hour period, how many breastfeeds does your baby have?									
Number of feeds directly from the breast (n = 20)	3a	0.92	47.4	0.41	0.86	26.7	0.20	6	0-12
Number of feeds of expressed breast milk (n = 17)	3b	0.84	91.7	0.85	0.58	75.0	0.57	0	0-8
Amount of expressed breast milk per feed (n = 12)	5	0.87	70.0	0.61	0.88	62.5	0.55	30 ml	0-250 ml
How many minutes does a typical daytime breastfeed directly from the breast last? (n = 19)	4	0.91	50.0	0.44	0.88	43.8	0.37	10 mins	0-30 mins
**Formula-feeding**									
In a typical 24 hour period, how much formula milk does your baby have?									
Amount of formula milk per feed (n = 26)	7a	0.74	52.2	0.43	0.79	58.3	0.47	210 ml	60-960 mls
Number of formula feeds per day (n = 25)	7b	0.57	73.9	0.68	0.58	45.5	0.37	4	1-8
How many minutes does a typical daytime bottle feed last? (n = 25)	8	0.81	63.6	0.54	0.94	52.2	0.44	15 mins	5-30 mins
Most of the time, how much milk is left in the bottle when your baby has finished feeding?									
none, a little, or a lot (n = 25)	9	0.84	91.3	0.78	0.63	83.3	0.57	72%- a little	
What was your baby's age in weeks when you first started formula feeds? (n = 26)	10	0.82	62.5	0.51	0.94	64.0	0.55	1.5 wks	0-15 wks
How many scoops of formula milk powder do you usually use per feed? (n = 25)	11d	0.73	73.9	0.62	0.64	70.6	0.52	7 scoops	2-8 scoops
**Other drinks**									
If your baby has water, how much water does your baby have?									
Amount of water per drink (n = 30)	13a	0.75	63.0	0.57	0.75	58.3	0.51	17.5 ml	0-150 ml
Number of drinks of water per day (n = 28)	13b	0.83	80.0	0.74	0.70	56.5	0.45	1	0-4
Does your baby have any drink other than milk or water? Yes OR No (n = 40)	14	0.88	97.4	0.87	0.72	91.7	0.68	90%- No	
**Solids/Semi-solids**									
Does your baby have any solid/semi-solid food? Yes OR No (n = 40)	15a	0.84	94.7	0.83	0.84	94.6	0.82	80%-Yes	
What was your baby's age in months when you started solid/semi-solid food? (n = 32)	15b	0.91	83.3	0.75	0.82	67.9	0.54	5 mo	4-6mo
How many times per day does your baby have solid/semi-solid food? (n = 32)	15c	0.63	73.3	0.63	0.60	65.5	0.49	3	1-4

^a^Test-retest reliability-1^st ^and 2^nd ^questionnaires

^b^Validity-1^st ^questionnaire and interview

^c^Question- Question number

^d^Corr-Spearman's correlation coefficient

^e^%Ag- Percentage exact agreement- over 66% good agreement

^f^Kap- Chance corrected agreement or Cohen's Kappa- < 0 poor; 0-0.20 slight; 0.21-0.40 fair; 0.41-0.60 moderate; 0.61-0.8 substantial; 0.81-1 almost perfect

Although percentage agreement between the two questionnaires was below 70% for six items (test-retest reliability), all 16 items in this domain had moderate to almost perfect agreement based on kappa values (above 0.41), and 80% (13/16) had kappa values above 0.41 for validity. The three items that had fair validity (Kappa 0.2-0.4) were continuous variables: number of breastfeeds, number of formula-milk feeds, duration of breastfeeds, and had correlation coefficients of 0.9, 0.6 and 0.9 respectively. It is unclear whether the questions are not valid or whether feeding practices changed over the duration between the first questionnaire and interview.

The median daily number of breastfeeds was six and the median duration of each feed was ten minutes. The median number of formula-milk feeds was four and median duration was 15 minutes. Eighty percent of mothers had introduced solid foods and 10% were giving drinks other than water and milk.

### General attitudes to infant feeding and growth (Table 3)

**Table 3:** Maternal attitudes to infant growth and feeding (9 items, n = 40)

Question Wording		**Test- Retest reliability**^**a**^		**Collapsed 3 categories**^**b**^		**Validity**^**c**^		**Frequency**^**d**^		
	Question	%agreement	Kappa	%agreement	Kappa	%agreement	Kappa	+ve	neutral	-ve
**Attitudes to growth**										
I think it is important to monitor the growth of my baby	16a	89.5	0.78	Insufficient variability in response				40	0	0
I would be worried if my baby was gaining too little weight	16b	94.7	0.84	100.0	1.00	93.2	0.48	34	6	0
I would be worried if my baby was gaining too much weight	16c	85.1	0.42	85.5	0.38	85.1	0.52	33	3	4
**Attitudes to feeding**										
It is possible to feed a baby too much	16e	90.8	0.69	89.5	0.78	73.6	0.47	20	4	16
It is possible to feed a baby too little	16f	92.1	0.73	89.5	0.74	70.8	0.44	28	3	9
**Self-efficacy to monitor growth**										
I am confident that I can get my baby measured if I was concerned about their growth	16d	82.9	0.36	97.4	0.00	97.3	0.00	39	1	0
**Self-efficacy for appropriate feeding**										
I am confident that I can feed my baby so they do not gain too much weight	16 g	91.5	0.57	89.5	0.60	82.4	0.00	29	8	3
I am confident that I can feed my baby so they gain enough weight	16h	92.8	0.42	93.4	0.50	91.9	0.00	36	2	2
**Perception of baby's weight**								under	right	over
Do you think your baby is underweight OR about right OR overweight	18	100.0	1.00	n/a	n/a	100.0	1.00	1	39	0

^a^Test-retest reliability-1^st ^and 2^nd ^questionnaires

^b^1^st ^and 2^nd ^Questionnaires coded as positive, or neutral, or negative

^c^Validity-1^st ^questionnaire and interview coded as positive, or neutral, or negative

^d^Frequency of respondents 1^st ^questionnaire

All items had percentage agreement over 70% for both reliability and validity. There was however not much variation in the responses hence kappa values were low for some of the items.

Seventy percent of the mothers agreed or strongly agreed with the statement that '*it is possible to feed a baby too little*'; whereas only 50% agreed or strongly agreed with '*it is possible to feed a baby too much*' (p = 0.002). Ninety percent were confident that they could feed their baby so that they gain enough weight; whereas 72% were confident that they could feed their baby so they do not gain too much weight (p < 0.001). Ninety-eight percent perceived that their baby's weight was about right.

### Theory-based beliefs about following recommendations to reduce formula-feed quantities (Table 4)

**Table 4:** Theory-based beliefs about following recommendations to reduce formula-milk feed quantities (11 items, n = 35)

Question Wording		**Test- retest reliability**^**a**^		**Collapsed 3 categories**^**b**^		**Validity**^**c**^		**Frequency**^**d**^		
	Question	%agreement	Kappa	%agreement	Kappa	%agreement	Kappa	+ve	neutral	-ve
**Positive outcome expectancy**										
If I follow the new feeding recommendation, my baby's growth will be optimal	19a	76.7	0.24	79.7	0.24	75.0	0.19	6	25	3
If I follow the new feeding recommendation, I will feel good about myself	19b	87.9	0.44	83.3	0.54	66.0	0.21	9	17	9
If I follow the new feeding recommendation, I will feel I do the best for my baby	19c	89.9	0.62	86.4	0.66	76.0	0.37	14	15	6
**Negative outcome expectancy (reverse coded)**										
If I follow the new feeding recommendation, my baby will remain hungry	19 g	89.1	0.40	79.7	0.33	70.8	0.32	8	22	3
If I follow the new feeding recommendation, my baby will wake up frequently at night	19h	91.4	0.47	84.4	0.44	68.0	0.31	6	24	4
**Positive self-efficacy**										
I am confident that I can follow the new feeding recommendation even if my baby cries between feeds	19d	91.9	0.76	90.9	0.77	76.0	0.37	6	12	17
I am confident that I can follow the new feeding recommendation even if my friends do not follow the same recommendation	19e	85.6	0.23	77.3	0.23	80.0	0.00	23	11	1
**Negative self-efficacy (reverse coded)**										
It would be difficult for me to follow the new feeding recommendation if my partner and family do not support me	19f	86.4	0.69	86.4	0.69	56.0	0.09	10	12	13
It would be difficult for me to follow the new feeding recommendation	19 k	89.1	0.45	82.8	0.51	68.0	0.27	10	17	7
**Intentions**										
I intend to follow the new feeding recommendation	19i	90.6	0.65	87.5	0.65	64.0	0.22	8	19	7
I will try to follow the new feeding recommendation	19j	92.7	0.75	92.2	0.81	70.0	0.32	14	12	8

^a^Test-retest reliability-1^st ^and 2^nd ^questionnaires

^b^1^st ^and 2^nd ^Questionnaires coded as positive, or neutral, or negative with negatively worded items reverse coded

^c^Validity-1^st ^questionnaire and interview coded as positive, or neutral, or negative with negatively worded items reverse coded

^d^Frequency of respondents 1^st ^questionnaire with negatively worded items reverse coded

The 11 items in this domain measured three constructs - Outcome-expectancies (5 items), Self-efficacy (4 items) and intentions (2 items). All items had percentage agreement over 76% for reliability and 82% (9/11) items had percentage agreement over 66% for validity. The two items that had percentage agreement below 66% (56% and 64%) had kappa values of 0.09 and 0.22.

As quantified by responses to the first questionnaire, mean self-efficacy score was 2.7 (median 3, inter quartile range 2.6-3.3); for outcome-expectancies mean score was 2.7 (median 3, inter quartile range 2.6-3.4); and for intentions mean score was 2.6 (median 3, inter quartile range 2-3.5), on a scale of 1-5. Internal consistency measured by Cronbach's alphas was 0.51, 0.79 and 0.90 for self-efficacy (4 items), outcome-expectancies (5 items) and intentions (2 items) respectively. Correlations between summary scores for self-efficacy, outcome-expectancy, and intentions were 0.84-0.92 (all p < 0.0001-Table 5),

**Table 5:** Correlation coefficients between summary score for self-efficacy, outcome-expectancies and intentions (n = 35)

	Outcome-expectancies	Intentions
**Self-efficacy**	0.92	0.84
**Outcome-expectancies**		0.85

## Discussion

We have developed a questionnaire to assess infant milk feeding practices and maternal attitudes. Test-retest reliability was good with 89% of the items having percentage agreement above 70% and the remaining six items having kappa values between 0.41-0.68 (moderate or substantial agreement). Comparing questionnaire with interview coding (criterion validity), percentage agreement was below 66% for 28% (16/57) of the items and of these five had kappa values below 0.20. We believe that this may have been due to the open-ended nature of the interview rather than the questionnaire which meant that it was not possible to code many of the interview responses. For example, in the interviews we asked '*How do you usually make up formula feeds', and 'have you received any advice on formula-feed preparation?' *Often it was not possible to code the questionnaires (in which very specific questions were asked) based on the interview responses.

The low internal consistency for four items measuring self-efficacy (Cronbach's alpha 0.5) was probably because although many mothers (23/35) were confident about following the infant feeding recommendations even if their friends did not follow them, fewer were confident about following the infant feeding recommendations if their partner or family did not support them (10/35), or if their baby cried between feeds (6/35) suggesting that the barriers may be quite distinct.

During the interview, mothers suggested that two of the items that asked about the possibility of feeding 'a' baby too much or too little could be reworded to specify 'my' baby (Question 16). They also said that the stem of question 19 which read '*New research suggests that babies should be given less formula milk than what is currently recommended on the formula milk packets/tins*...'was difficult to answer without knowing how much lower the new recommendations were. We have changed this in the current version of the questionnaire to read '*We would like to know your views on following the feeding recommendations available to you, on how much milk your baby needs...'*. Since these two questions have been changed (question16 and 19), future studies will have to confirm their validity in a larger sample to explore the underlying factor structure.

While all of the mothers thought that it was important to monitor the growth of their babies, only half of them thought that it was possible to feed a baby too much. Among the mothers who prepared formula-milk feeds from powder, forty percent tightly packed the scoops. These attitudes could contribute to higher growth rate and obesity levels among formula-milk fed babies by overconcentration of milk feeds.

## Strengths and limitations

The main strengths of this study are that the participants were mothers of infants using a variety of feeding methods (exclusive breastfeeding, exclusive formula-milk feeds and a combination). The validity and reliability are comparable to other questionnaires measuring attitudes and behaviours[19,35]. The questions on frequency and quantity of milk feeds can be administered frequently during the first six months of life (in large-scale intervention and observational studies) to measure energy intake and to study the association between energy intake and infant growth. The questions on attitudes and theory-based beliefs can be used to study differences between populations and to assess intervention mediation pathways in obesity prevention trials starting in early life.

The time interval between two questionnaires was approximately one week and reliability may be lower if the time interval is longer. However since feeding practices change very frequently during infancy, we did not think that it would be appropriate to have a longer time interval because we could be measuring actual change in feeding practices.

Although our preliminary validation suggests that the tool could be useful, gold-standard psychometric methods to comprehensively evaluate the tool in a larger sample and different populations will be necessary to confirm acceptability, reliability and validity[36]. Another limitation of the small sample size was that it was not possible to explore important issues such as the influence of feeding behaviours, timing of completion of questionnaires and participant demographics. Many mothers participating in the study were 'White', had 'degree' level qualifications and a 'professional' occupation which may have influenced our findings. Ten percent of mothers were exclusively breastfeeding and data from the UK National Infant Feeding Survey suggest that 1% of mothers exclusively breastfed at six months[37].

## Conclusions

We have developed a questionnaire to measure milk-feeding practices and maternal attitudes to feeding and growth in early life. This questionnaire could be a useful tool to provide much needed insight in the 'causal mechanism' of interventions that target infant feeding practices to prevent early obesity.

## Funding

The study was supported by the Medical Research Council and the Centre for Diet and Activity Research (CEDAR), a UKCRC Public Health Research Centre of Excellence, which is funded by the British Heart Foundation, Department of Health, Economic and Social Research Council, Medical Research Council, and the Wellcome Trust, under the auspices of the UKCRC. RL is supported by a MRC Health Services and Health of the Public Research Fellowship and is in receipt of a Raymond and Beverley Sackler fellowship.

## Competing interests

The authors declare that they have no competing interests.

## Authors' contributions

RL developed the questionnaire with input from all co-authors. RL and JL conducted the interviews. RL analysed the data and wrote the first draft. All authors contributed to the study design, critical revision of the manuscript, and approved the final version.

## Ethical Approval

Cambridgeshire 1 Research Ethics committee 09/H0304/25 and Essex 2 Research Ethics committee 08/H0302/47.

## Supplementary Material

Additional file 1 Interview schedule Click here for file
