# Supplementary material for: Development of a questionnaire to assess maternal attitudes towards infant growth and milk feeding practices
Source: Int J Behav Nutr Phys Act. 2011 Apr 21;8:35. doi: 10.1186/1479-5868-8-35 (PMC3111341; doi:10.1186/1479-5868-8-35)
Supplement: Additional file 1 — Interview schedule [file 1479-5868-8-35-S1.DOC]

**Additional file 1: Interview schedule**

**Introduction**

Hello my name is *staff name*. I work on the *Baby Growth/Baby Milk* study. Is *participant name* available?

Hi *participant name*. Is this a convenient time to talk for about half an hour?

Thank you for filling in the questionnaires. Hope you don’t mind having to do them twice (check that participant has filled out both questionnaires and sent them back). This is our way of checking that the questions were clear and easy to understand.

Can I put on the recording machine and go through the questions one more time?

(If says ‘yes’- switch on the recorder).

Before we go through the questions, did you have any comments on the questionnaire?

Thank you

1. What feeding methods are you currently using?

2. How do you decide how often (or when) to feed your baby?

**If mum breastfeeds ask questions 3-5 next. If formula feeds, move onto question 6.**

3. How many breastfeeds or feeds of expressed breast milk does your baby have in a 24 hour period?

4. How many minutes does a typical daytime breastfeed directly from the breast last?

5. Do you give your baby any expressed breast milk? how often and how much expressed milk does your baby usually drink per feed?

**If mum formula feeds ask questions 6-12. If no, move on to question 13.**

6. What brand and type of formula do you usually use?

7. How many formula feeds does your baby have per day? How much formula do you use per feed?

8. How many minutes does a typical daytime bottle feed last?

9. How much milk is left in the bottle when your baby has finished feeding?

10. How old was your baby when you first started formula feeds?

11. Could you please tell me how you usually make the formula milk? (for example how you fill the scoops, do you add powder or water first to bottle, how many scoops per feed)

12. Have you received advice on how to prepare the formula? From whom/where? Which advice did you follow?

**I would like to now ask you a few questions regarding your baby’s other feedings.**

13. Do you give your baby water? If yes, how many drinks of water does your baby have per day? How much water at a time is given to your baby?

14. Does your baby have anything else to drink other than milk or water?

15. Does your baby have any solid/semi-solid foods? If yes, what was your baby’s age in months when you started solid/semi-solid foods?

How many times per day does your baby have solid/semi-solid foods?

**I would like to now ask you a few questions regarding your views on your baby’s growth and feeding.**

16. How do you feel about monitoring your baby’s growth?

How would you feel if your baby was gaining too little weight?

How would you feel if your baby was gaining too much weight?

How confident are you in monitoring your baby’s growth through having him/her measured?

How do you feel about the possibility of feeding your baby too much?

How do you feel about the possibility of feeding your baby too little?

How confident are you in feeding your baby just right so that they do not gain too much weight or too little weight?

17. How do you decide how much to feed your baby?

18. Currently, how would you describe your baby’s weight?

**Thank you so much for your time thus far. We have one last section regarding the new formula feeding recommendations which suggest that babies should be given less formula milk. I would like to ask you questions regarding your views on these recommendations.**

How do you feel these recommendations will affect the growth of your baby?

How would you feel about yourself if you followed the new recommendations?

How would feel about following the recommendations in order to do the best for your baby?

How confident do you feel that you can follow the new guidelines even if your baby is crying between feeds?

How confident do you feel that you can follow the new guidelines even if your friends are not following them?

How difficult would it be too follow the new recommendations if your partner and/or family did not support you?

How do you feel these recommendations will affect your baby’s hunger?

How do you feel these recommendations will affect your baby’s sleeping at night?

Do you think you would follow new recommendations?

How do you feel about trying to follow the new recommendations?

How difficult do you think it will be for you to follow the new feeding recommendations?

That completes the questions. Thanks again *name* for your willingness to help us with these questions. I hope I haven’t taken too much of your time.

Have a good day.
